# Renal Cell Carcinoma with Testicular Metastases: A Case Report and Review of the Literature

**DOI:** 10.15586/jkcvhl.v9i2.212

**Published:** 2022-05-06

**Authors:** Sho Yoshitake, Brian M. Shinder, Kevin Dazen, Colton Smith, Tina M. Mayer, Evita Sadimin, Eric A. Singer

**Affiliations:** 1 Section of Urologic Oncology, Rutgers Cancer Institute of New Jersey and Rutgers Robert Wood Johnson Medical School, New Brunswick, NJ, USA;; 2 Department of Pathology, Rutgers Cancer Institute of New Jersey and Rutgers Robert Wood Johnson Medical School, New Brunswick, NJ, USA;; 3 Division of Medical Oncology, Rutgers Cancer Institute of New Jersey and Rutgers Robert Wood Johnson Medical School, New Brunswick, NJ, USA;; 4 Department of Pathology, City of Hope National Medical Center, Duarte, CA, USA

**Keywords:** clear cell, metastatic renal cell carcinoma, renal cell carcinoma, systemic therapy, testicular mass

## Abstract

Renal cell carcinoma (RCC) metastases to the testicle are an extremely rare clinical entity. Here, we describe the case of a man with metastatic RCC who developed a new testicular mass. Pathologic analysis after surgical removal of this testicle confirmed the diagnosis of metastatic RCC. This report highlights the unique diagnostic and therapeutic challenges associated with such a disease process.

## Introduction

The incidence of renal cell carcinoma (RCC) in the United States has been steadily rising over the past decades (1). In 2021, there will be approximately 76,000 new cases and 16,000 deaths due to RCC (2). Historically, RCC was associated with the triad of flank pain, hematuria, and palpable abdominal mass. Current estimates, however, suggest that only 9% of patients present with all these symptoms, and their presence likely signals an advanced disease state (3). More commonly, an incidental mass is found by either ultrasound or CT for an unrelated problem.

Local extension of RCC into the renal capsule, renal sinus, or collecting system occurs in approximately 20% of cases, with advanced disease progressing past the protective layer of Gerota’s fascia (4). A unique feature of RCC is its natural preference for venous system involvement. RCC tumors may extend intraluminally in the renal venous circulation, with cephalad inferior vena cava (IVC) migration, and renal vein or IVC tumor thrombus will be present in up to 10% of the patients (5). For most patients, RCC remains an organ-confined disease, and surgical resection results in excellent survival outcomes (6, 7). However, there are still treatment challenges in those with advanced or metastatic stage of the disease (8). Approximately 25–30% of patients present with metastatic disease, while 20–40% of men and women who undergo surgical resection for localized RCC will develop metastases (9, 10).

In patients with metastatic disease, prognosis depends upon many factors, including the number and location of metastatic sites (11, 12). Although metastatic spread is common in the lungs, bone, distant lymph nodes, and liver, atypical sites are occasionally involved (11, 13). Various reports have described the spread of RCC to the head, neck, skin, skeletal muscle, and pelvis (14). Given the relative rarity of these, much less is known about their optimal treatment pathways. Additionally, metastatic spread of RCC to atypical sites may mimic other clinical entities, presenting a diagnostic challenge (15). Here, we report the case of a patient who developed metastatic RCC of his right testicle following radical nephrectomy for localized disease. All potential patient identifiers have been removed, conforming with Institutional Review Board exemption standards.

## Case Report

A 63-year-old man who was being followed with serial abdominal ultrasounds for benign prostatic hyperplasia was found to have a right renal mass, several years prior to presentation at our institution. He reported no systemic symptoms at the time of the finding and underwent a right radical nephrectomy. Pathology was not available for this as the surgery was performed in the patient’s home country. The patient was followed with serial CTs, and 3 years after initial radical nephrectomy, he was found to have a 4.6 cm right thoracic paraspinal mass along with parenchymal nodules, 5.7 mm in the right upper lobe and 7 mm in the left lower lobe. He denied any symptomology at this time.

**Figure 1:**
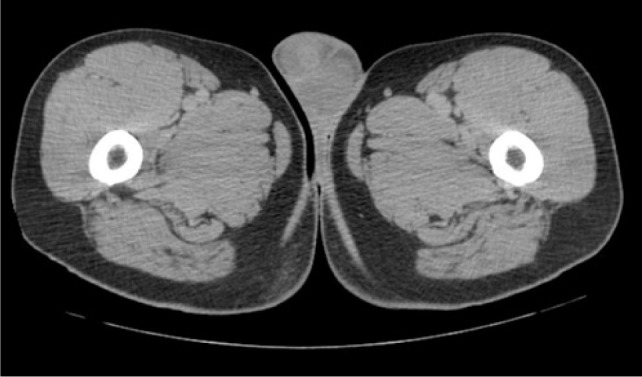
CT urogram showing 1.7-cm enhancing mass in the lower pole of right testicle.

**Figure 2:**
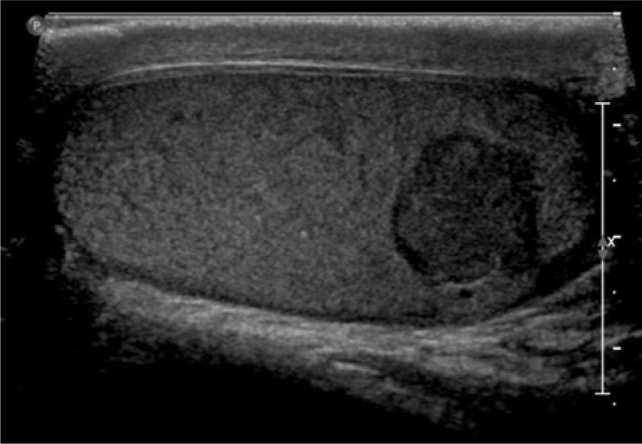
Ultrasound image showing 1.4 × 1.5 × 1.5 cm hypoechoic mass in the lower pole of right testicle.

Core biopsy confirmed metastatic clear cell RCC in the paraspinal mass. The mediastinal and bilateral parenchymal masses were resected. Pathology for the mediastinal and left lung masses was positive for clear cell RCC, while hamartoma was confirmed in the right lobe. A PET/CT done 1 year later, showed suspicious uptake in the adrenal lesions being monitored. The right mass had a standardized uptake value (SUV) of 2.7, while the left mass had an SUV of 2.9. Labs showed normal levels of aldosterone, cortisol, plasma metanephrines, and normetanephrines. Biopsy of the right adrenal mass revealed pathology consistent with metastatic RCC. After an initial period of surveillance, the patient chose to proceed with systemic therapy and enrolled on a clinical trial. He had stable disease for 2 years when a CT urogram revealed a 1.7-cm enhancing right lower pole testicular mass (Figure 1). Physical examination revealed a palpable right lower pole testicular mass. Testicular cancer serum tumor markers were within normal limits. Ultrasound showed a 1.5 cm hypoechoic, hypervascular right lower pole mass (Figure 2). CT scans showed stability of prior metastatic lesions.

**Figure 3:**
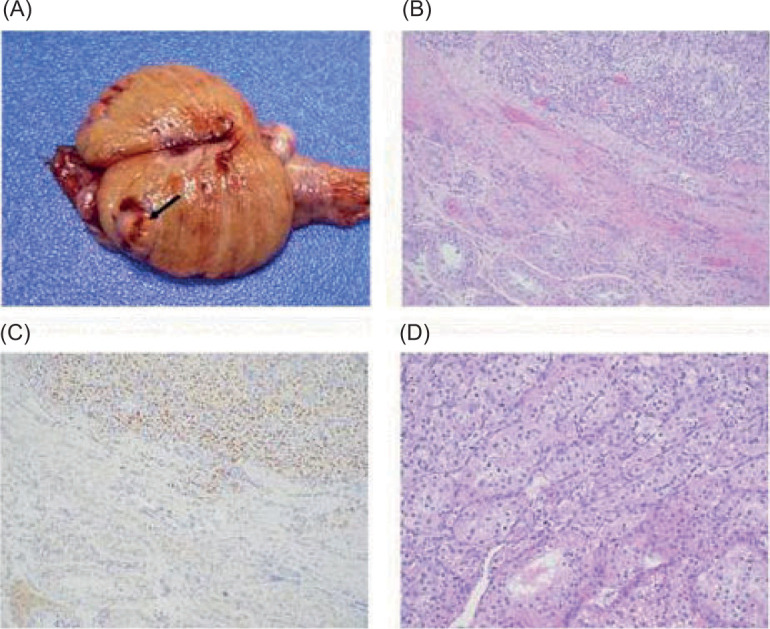
Gross and microscopic pathology of right radical orchiectomy specimen. (A) Well-circumscribed golden orange lesion (arrow) confined to the testicular parenchyma. (B) Solid nests of tumor (top right) separated by fibrous band adjacent to seminiferous tubules (bottom left) (100x magnification). (C) By immunohistochemistry, the tumor is positive for PAX8 (100x magnification). (D) Under higher magnification, the tumor cells show clear cytoplasm and prominent nucleoli (200x magnification).

A right radical orchiectomy via an inguinal approach was performed, and pathology showed metastatic clear cell RCC. On macroscopic examination, it was revealed that the lesion involved testicular parenchyma, measuring 1.3 cm in greatest dimension. Microscopically, the tumor displayed the typical histology of clear cell RCC, consisting of tumor cells arranged in solid nests with clear cytoplasm, surrounded by fine capillaries. These cells were positive for PAX8, further confirming renal origin (Figure 3).

After almost 20 months of follow-up from his radical orchiectomy, the patient has an overall stable appearing disease on imaging studies and continues to undergo routine surveillance.

## Discussion

Metastatic spread of solid tumors to the testes is exceedingly rare. The testes are considered a “tumor sanctuary,” as the low temperature in the scrotum provides an inhospitable environment for metastatic cells (16). Furthermore, the blood–testis barrier, which protects spermatozoa from targeting by the body’s immune system, may have an indirect role in preventing metastasis to the testis (17). In an autopsy study of 738 autopsies of adult males with solid neoplasms, five (0.68%) showed metastases to the testis (18). Most commonly, the spread occurs from the prostate, accounting for nearly half of the cases of testicular metastases (16). In 11,157 patients with metastatic RCC, from 1998 to 2007, the most common sites of spread were to the lung (45.2%), bone (29.5%), distant lymph node (21.8%), and liver (20.3%); metastases to the testes were not noted in this study (13). To our knowledge, there have been less than 50 reported cases of RCC metastasis to the testis (17, 19–27).

A review of the available literature seems to suggest that metastatic spread to the ipsilateral testis is more common than contralateral or bilateral spread (17, 19–27). It has been hypothesized that metastasis occurs by retrograde venous spread, especially considering the anatomic relationship between the left renal and gonadal veins (28). To account for contralateral and bilateral metastases, it has been suggested that metastases might spread by Batson’s venous plexus (23). Other hypotheses include arterial and lymphatic involvement, as well as iatrogenic seeding (25, 29). However, the full mechanism driving metastases to the testes has still not been fully elucidated.

RCC metastasis to the testis is difficult to diagnose, as considerable heterogeneity exists among prior cases. In symptomatic patients, scrotal enlargement and presence of a testicular mass are two common symptoms (17, 24, 27). However, metastatic carcinomas to the testes are most commonly detected incidentally during autopsy (30). Metastases to the testis have also been diagnosed, both prior to initial treatment of RCC and up to 7 years following treatment (17, 19–27). Metastatic burden is also variable, as patients ranged from having a solitary testicular metastasis to widespread involvement of multiple organs (17, 19–27).

Due to the rarity of RCC metastasis to the testis, the clinical suspicion for it may be quite low. In this regard, difficulty distinguishing an RCC metastasis to the testes from primary testicular tumors also adds to the challenges in diagnosing metastasis. Testicular cancer represents about 1–2% of all cancers in males, with around 9,400 new cases reported in the United States in 2021 (2, 31). Though it mostly occurs in younger patients and is the most common malignancy among males aged 15–40, around 8% of cases occur in patients aged >50 (32–34). Furthermore, diagnosis at age >50 is associated with a lower 10-year relative survival (33). As such, primary testicular cancer should not be ruled out simply based on advanced age. In the setting of a unilateral scrotal mass, ultrasound imaging is recommended to further characterize the testicles, and serum tumor markers are typically checked (35, 36). However, RCC metastasis to the testis has similar ultrasonographic findings as primary testicular tumors, and serum tumor markers may be negative in both (21, 25, 36).

For suspected testicular cancers, pathologic diagnosis is obtained by a radical orchiectomy via an inguinal approach. Partial orchiectomy is typically reserved for selected cases such as bilateral testicular cancer and germ cell tumors in patients with solitary testis (37). This approach may confer some clinical benefits, including a decreased need for hormone replacement, improved psychological outcomes, and preservation of fertility (37). In theory, for cases where RCC metastasis to the testis is suspected, partial orchiectomy might be considered. Nonetheless, radical orchiectomy is likely necessary for a definitive diagnosis and to prevent deviations in standard of care for a patient with a potential testicular cancer, given the difficulty in discerning between a primary testicular tumor and a metastatic lesion.

Optimal treatment pathways for metastatic RCC are yet to be completely defined. Metastasectomy has been associated with longer overall survival (OS) and cancer-specific survival (CSS) compared to incomplete and/or no metastasectomy (38). Targeted systemic therapy with vascular endothelial growth factor tyrosine kinase inhibitors (VEGF-TKIs), such as sunitinib and pazopanib, has been recommended for most patients with metastatic RCC (39). However, emerging immune oncology (IO) agents targeting immune checkpoint pathways such as PD-L1 and CTLA-4 have changed the landscape of metastatic RCC treatment. Additionally, combination regimens of IO agents together or with VEGF-TKIs have been shown to be more efficacious than single agents (39, 40). Further investigations on the role of surgical resection of metastatic sites in conjunction with systemic therapy are certainly warranted, especially in the setting of rarer metastatic sites such as the testes.

## Conclusion

RCC imparts a large burden on health globally. This report highlights an interesting case of metastatic RCC to the testes. Although exceedingly rare, metastatic RCC to the testes presents a unique diagnostic and therapeutic challenge. Consideration for this disease entity is warranted in anyone with a history of RCC and a new testicular mass, though providers must also have a high index of suspicion for a primary testicular cancer.
